# Validating a Rapid, Automated Test of Spatial Release From Masking

**DOI:** 10.1044/2017_AJA-17-0013

**Published:** 2017-12-12

**Authors:** Kasey M. Jakien, Sean D. Kampel, Meghan M. Stansell, Frederick J. Gallun

**Affiliations:** a National Center for Rehabilitative Auditory Research, VA Portland Health Care System, U.S. Department of Veterans Affairs, OR; b Department of Otolaryngology/Head & Neck Surgery, Oregon Health & Science University, Portland

## Abstract

**Purpose:**

To evaluate the test–retest reliability of a headphone-based spatial release from a masking task with two maskers (referred to here as the SR2) and to describe its relationship to the same test done over loudspeakers in an anechoic chamber (the SR2A). We explore what thresholds tell us about certain populations (such as older individuals or individuals with hearing impairment) and discuss how the SR2 might be useful in the clinic.

**Method:**

Fifty-four participants completed speech intelligibility tests in which a target phrase and two masking phrases from the Coordinate Response Measure corpus (Bolia, Nelson, Ericson, & Simpson, 2000) were presented either via earphones using a virtual spatial array or via loudspeakers in an anechoic chamber. For the SR2, the target sentence was always at 0° azimuth angle, and the maskers were either colocated at 0° or positioned at ± 45°. For the SR2A, the target was located at 0°, and the maskers were colocated or located at ± 15°, ± 30°, ± 45°, ± 90°, or ± 135°. Spatial release from masking was determined as the difference between thresholds in the colocated condition and each spatially separated condition. All participants completed the SR2 at least twice, and 29 of the individuals who completed the SR2 at least twice also participated in the SR2A. In a second experiment, 40 participants completed the SR2 8 times, and the changes in performance were evaluated as a function of test repetition.

**Results:**

Mean thresholds were slightly better on the SR2 after the first repetition but were consistent across 8 subsequent testing sessions. Performance was consistent for the SR2A, regardless of the number of times testing was repeated. The SR2, which simulates 45° separations of target and maskers, produced spatially separated thresholds that were similar to thresholds obtained with 30° of separation in the anechoic chamber. Over headphones and in the anechoic chamber, pure-tone average was a strong predictor of spatial release, whereas age only reached significance for colocated conditions.

**Conclusions:**

The SR2 is a reliable and effective method of testing spatial release from masking, suitable for screening abnormal listening abilities and for tracking rehabilitation over time. Future work should focus on developing and validating rapid, automated testing to identify the ability of listeners to benefit from high-frequency amplification, smaller spatial separations, and larger spectral differences among talkers.

Research has shown that the audiogram alone may not be the most accurate predictor of who may struggle with speech in competition (Ruggles, Bharadwaj, & Shinn-Cunningham, 2011; Strelcyk & Dau, 2009) despite the fact that the audiogram is the most commonly used test in the modern audiology clinic. Indeed, speech testing, when done, is almost always conducted in quiet, due primarily to limitations on clinical testing time. Furthermore, there is little appreciation of the benefits for diagnosis and rehabilitation that would be derived from testing speech in any type of competition. Nonetheless, one of the most common difficulties for people with hearing impairment is the reduced intelligibility of speech in complex acoustical environments (Humes & Dubno, 2010). An important aspect of speech understanding in complex environments, which is even more rarely explored clinically, is the ability to use spatial separations between sound sources to achieve spatial release from masking (SRM). Listeners vary substantially in performance on speech-in-speech tasks (Swaminathan et al., 2015), but in general, they attain better thresholds when sound sources are spatially separated rather than colocated, in part due to access to binaural cues such as interaural differences in time (ITD) and level (ILD) (Bronkhorst, 2015). While most listeners with normal hearing (NH) achieve substantial SRM with even fairly small separations, older listeners and listeners with hearing impairment (OHI/HI) generally derive less benefit from spatial separation (Ahlstrom, Horwitz, & Dubno, 2014; Besser, Festen, Goverts, Kramer, & Pichora-Fuller, 2015; Dubno, Dirks, & Morgan, 1984; Ellinger, Jakien, & Gallun, 2017; Gallun, Diedesch, Kampel, & Jakien, 2013; Helfer & Freyman, 2008; Marrone, Mason, & Kidd, 2008; Srinivasan, Jakien, & Gallun, 2016).

Besser et al. (2015) studied the importance of high-frequency information on SRM for younger and older listeners and found that for both groups, spatial advantage was predicted by a high-frequency pure-tone average (PTA; 6–10 kHz). This suggests that the amount of hearing impairment in the high-frequency regions might predict SRM. One way that listeners might use information in the high-frequency region of the speech stimuli to improve performance in the presence of speech maskers could be by using the frequent low-energy periods in high-frequency portions of the masker to “glimpse” the target (Ahlstrom et al., 2014; Best et al., 2016; Best, Mason, Swaminathan, Roverud, & Kidd, 2017; Brungart & Iyer, 2012; Cooke, 2006; Glyde, Buccholz, Dillon, Best, et al., 2013). Other researchers have found that speech intelligibility can improve with access to high-frequency information, either for aided or unaided listeners (Ahlstrom, Horwitz, & Dubno, 2009; Ahlstrom et al., 2014; Levy, Freed, Nilsson, Moore, & Puria, 2015; Moore, Füllgrabe, & Stone, 2010) and for both listeners with NH and with HI without hearing aids (Jakien, Kampel, Gordon, & Gallun, 2017; Levy et al., 2015). Ellinger et al. (2017) found that ITD and ILD are both important for SRM, suggesting that low-frequency audibility is also likely to contribute to the release obtained.

The effects of aging on SRM as distinct from hearing loss are less clearly shown in the literature, especially as age and hearing loss nearly always co-vary, at least to some extent. For example, Marrone et al. (2008) found a negative correlation between age and SRM but did not have the statistical power to distinguish the smaller age effects from the larger effects of hearing loss on SRM. Similarly, Glyde, Cameron, Dillon, Hickson, and Seeto (2013) examined listeners varying over a large age range (7–89 years) with a wide range of hearing losses and did not find a significant effect of age on speech comprehension. To address this, Gallun et al. (2013) recruited participants in such a way that age and hearing thresholds were only weakly related and were able to show strong age effects on SRM. Similarly, Srinivasan et al. (2016), based on the hypothesis that older listeners are less sensitive to interaural correlation than are younger listeners, used smaller separations between target and masker than are typically used to try to find evidence of aging effects on SRM. Stepwise multiple linear regression showed that there were significant age effects at 4° and 6°, whereas at 8° onward, there was a strong effect of PTA, supporting the idea that in conditions with very small spatial separations between competing sound sources, there is an effect of age that is overshadowed by PTA as separations increase. Despite this evidence, however, it is still unclear whether these age effects reflect a reduced ability to separate competing sound sources due to cognitive processing impairments (Füllgrabe, Moore, & Stone, 2015; Schneider, Pichora-Fuller, & Daneman, 2010) or due to age-related degradation of the peripheral and/or central auditory pathways as have been observed in animal models of aging (Helfert, Sommer, Meeks, Hofstetter, & Hughes, 1999; Sergeyenko, Lall, Liberman, & Kujawa, 2013).

Some have also suggested (Dillon, Cameron, Glyde, Wilson, & Tomlin, 2012) that reduced SRM can be a sign of a central auditory processing disorder ([C]APD), which can occur both in younger (Cameron & Dillon, 2008) and older patients (Martin & Jerger, 2005). Issues arising from (C)APD may include trouble hearing speech when multiple people are talking, difficulty following rapid speech, and the inability to localize accurately. An audiologist diagnoses (C)APD with a large battery of tests that can include assessments of SRM. However, as clinical sessions are time constrained, it would be to the advantage of the audiologist to have a short, automated test to determine release from masking. The currently existing tests, such as the Listening in Spatialized Noise–Sentences Test (LiSN-S; Cameron & Dillon, 2007), are rapid and reliable but are not entirely automated as they require scoring by the administrator and, thus, may not be ideal for a busy clinical practice. Given that most clinical testing does not even include speech testing in noise, it would be of great benefit to have access to a rapid measure of performance that could be administered while the patient was waiting to be seen without the need for a clinician to be present during the administration of the test. This would provide additional information about the abilities of a given patient or participant that would not normally be able to be obtained without reducing the time available for other test measures.

To provide the tools necessary for automated SRM testing to be incorporated into the lab or clinic requires that reliable tests be both available and easy to use. Here, we answer several questions related to an automated test that is currently freely available and evaluates SRM without the need for scoring by an administrator. This test is referred to as the SR2, which indicates that it is a test of spatial release with two maskers. The SR2 can be run in an automated fashion over headphones with the potential to be delivered via a personal computer or tablet. Calibration routines have been included that can be performed for a wide range of hardware configurations. The SR2 examines SRM at 45°, which captures group differences in performance for which age and hearing loss can still be distinguished (Gallun et al., 2013). The test can be implemented to assess speech-in-speech masking and uses sentences from the Coordinate Response Measure corpus (CRM; Bolia et al., 2000), which has been utilized in over 50 studies to assess speech-in-speech intelligibility (e.g., Arbogast, Mason, & Kidd, 2002; Best, Gallun, Ihlefeld, & Shinn-Cunningham, 2006; Brungart, 2001; Humes, Lee, & Coughlin, 2006; Ihlefeld & Shinn-Cunningham, 2008; Johnsrude et al., 2013; Kidd, Arbogast, Mason, & Gallun, 2005; Li & Loizou, 2008; Mesgarani & Chang, 2012; Rossi-Katz & Arehart, 2009; Singh, Pichora-Fuller, & Schneider, 2008; Wightman, Kistler, & Brungart, 2006).

To capture the reliability of the SR2, we examined how participant performance changes over several testing sessions. To examine validity, we compared the headphones-based SR2 with the same test done over loudspeakers in an anechoic chamber (the SR2A). To demonstrate the value of this test with a clinical population, we tested listeners varying in age and hearing ability.

## General Method

In all tests, participants were asked to identify the target CRM phrase spoken by a male talker (the color–number combination after the call sign “Charlie”, always at 0° azimuth angle) in the presence of two masking sentences that were also spoken by male talkers. The masker and target talkers varied from trial to trial. Three talkers total were used. The fourth male talker in the corpus was not used due to his slower rate of speaking. Maskers could be either colocated with the target at 0° or symmetrically offset to the left and right by one of a variety of spatial separations. Responses were obtained using a monitor located in front of the participant showing an interface displaying all color and number options. Participants initiated each track and were given feedback (“Correct” or “Incorrect”) after each answer.

A progressive tracking procedure was used to present 20 trials, two at each of 10 target-to-masker ratios (TMRs), starting at 10 dB and ending at −8 dB (decreasing in steps of 2 dB). Because performance always starts near perfect and ends near chance and there are two presentations at each 2-dB step, the number of correct responses can be used to estimate the point at which performance is near 50% correct. Thus, TMR thresholds in decibels were estimated by subtracting the number of correct responses from the starting TMR value of 10 dB. As an example, if a listener correctly reported all of the trials at TMR values greater than 2 dB, but none below this value, eight trials would be scored correctly, leading to a threshold estimate of 2 dB. The difficulties with this method occur when the TMR values presented do not cover the range between very good and very poor performance (Gallun et al., 2016). Comparison with adaptive methods (Gallun et al., 2013) suggests that these difficulties do not interfere with the use of this test for the evaluation of patients. SRM was determined as the difference between thresholds in the colocated condition and the spatially separated conditions.

The SR2 involved headphone presentation in which target and masking speech were presented over Etymotic ER2 insert earphones. The target was always at 0° azimuth angle, and the maskers were colocated or positioned at ± 45°. Head-related impulse responses were convolved with the target and maskers to simulate the colocated and spatially separated target and masking speech conditions as described in Gallun et al. (2013). The use of equal sensation levels was achieved by first measuring the speech reception threshold (SRT) for each listener, using standard audiometric methods. This value was then transformed to obtain a uniform presentation level for the target of 39.5 dB SL. The two masker sentences were presented at levels relative to the target and were appropriately scaled in SL. No listeners were tested for whom maskers would have exceeded 85 dB SPL.

The SR2A, which stands for testing of the SR2 in an anechoic chamber, tested six spatial configurations: colocated, ± 15°, ± 30°, ± 45°, ± 90°, and ± 135°. As in the SR2, one target sentence and two masking sentences from the CRM were presented, and only the three male talkers mentioned above were used. All sentences were presented via calibrated loudspeakers hung in a circular arc at a distance of 1.5 m from the listener. Listeners completed four runs of each of the six spatial conditions and input their responses using the same method as in the SR2. Rather than relying upon the SRT to calculate the sensation level, the first test session started with an estimate of the just audible level for CRM stimuli, which was defined as the level supporting 50% identification of a male target presented in quiet at 0° azimuth. This level was obtained using a single run of a one-up/one-down adaptive track with two reversals at a step size of 4 dB, which were discarded, and a subsequent set of six reversals at 2 dB, which were averaged to estimate the just audible level. Stimuli were presented at 30 dB above this just audible level.

Over a time period of 48 months (November 2012–November 2016), over 100 participants completed between 1 and 15 runs of the SR2 at the National Center for Rehabilitative Auditory Research in Portland, Oregon. Data from 23 of those participants are described in Gallun et al. (2013), on the basis of their first two runs. Twenty-nine of these 100 individuals completed the SR2A four times in a single test session and the SR2 at least two times, 20 of whom also participated in Gallun et al. (2013). Experiment 1 analyzes these data for test-reliability and as a way of comparing performance on the SR2 and the SR2A. Experiment 2 examines test–retest reliability by comparing performance on the SR2 for those 40 of the 100 total listeners who repeated the SR2 at least eight times. Data from the listeners not discussed here will be analyzed in a subsequent report focusing on estimating normative functions on the basis of age and hearing thresholds. Procedures were approved by the VA Portland Health Care System Institutional Review Board, and all participants were monetarily compensated for their time.

## Experiment 1

### Subjects

**Figure 1.**
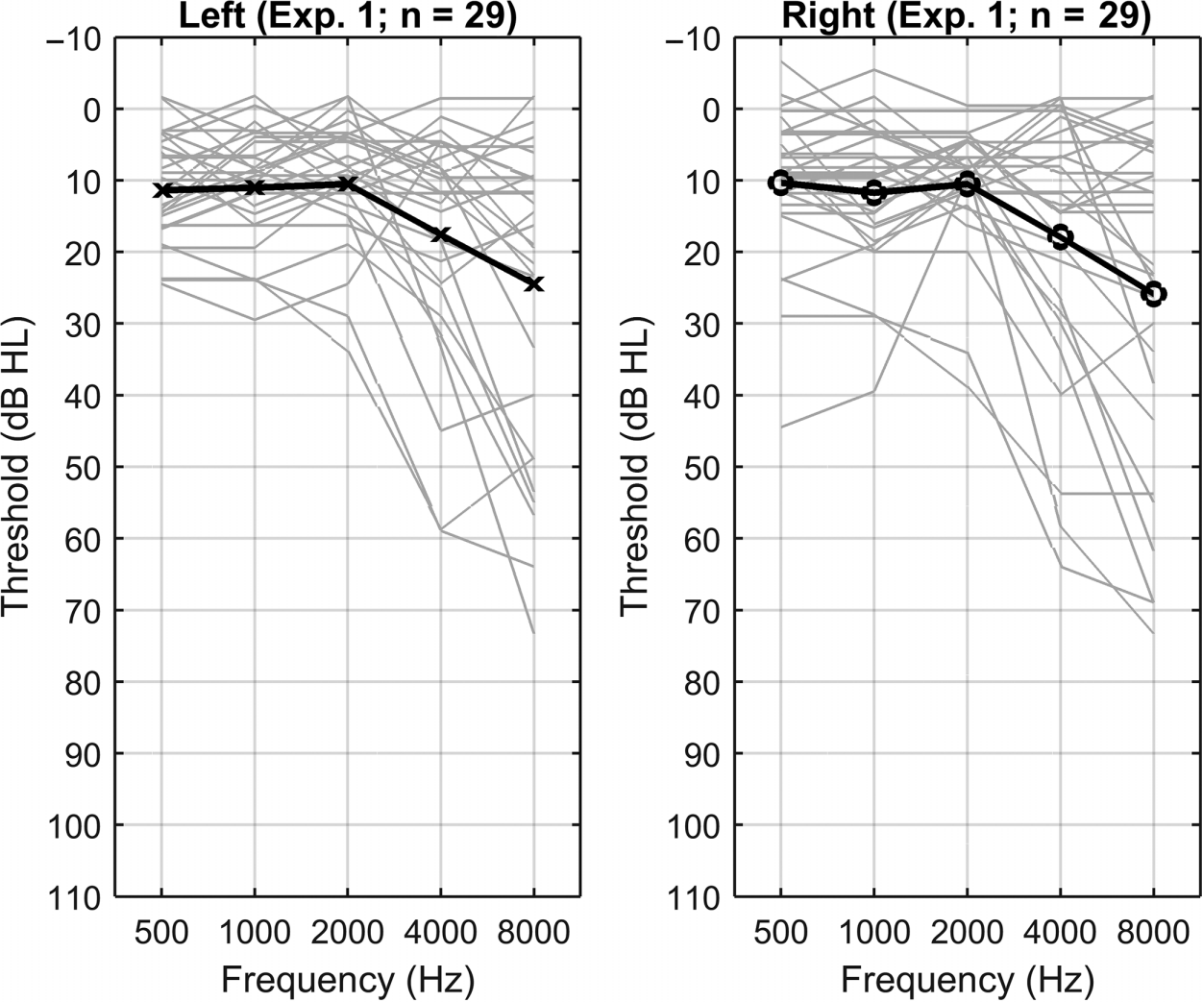
Mean (black lines) and individual (gray lines) audiometric thresholds for the 29 individuals from Experiment 1.

Participants were aged 21–77 years (mean 46.14 years), and all 29 had standard bilateral PTAs (average of 0.5, 1, and 2 kHz) below 30-dB HL (mean 10.92 dB ± 6.77-dB HL) and high-frequency PTAs (average of 2, 4, and 8 kHz) below 57.50-dB HL (mean 20.04 dB ± 1.41-dB HL). SRT values ranged from 0 to 30 dB HL. All participants had fairly symmetrical hearing at 2 kHz and below (most had differences between the ears of less than 10 dB at all frequencies, and none had differences exceeding 20 dB). Average audiograms are shown in Figure 1. All listeners were in good health with no history of otological disorders, had scores of 24 or higher on the Mini-Mental State Examination (Folstein, Folstein, & McHugh, 1975) to rule out dementia or any other cognitive impairments, and none of the individuals used hearing aids.

### Results

**Figure 2.**
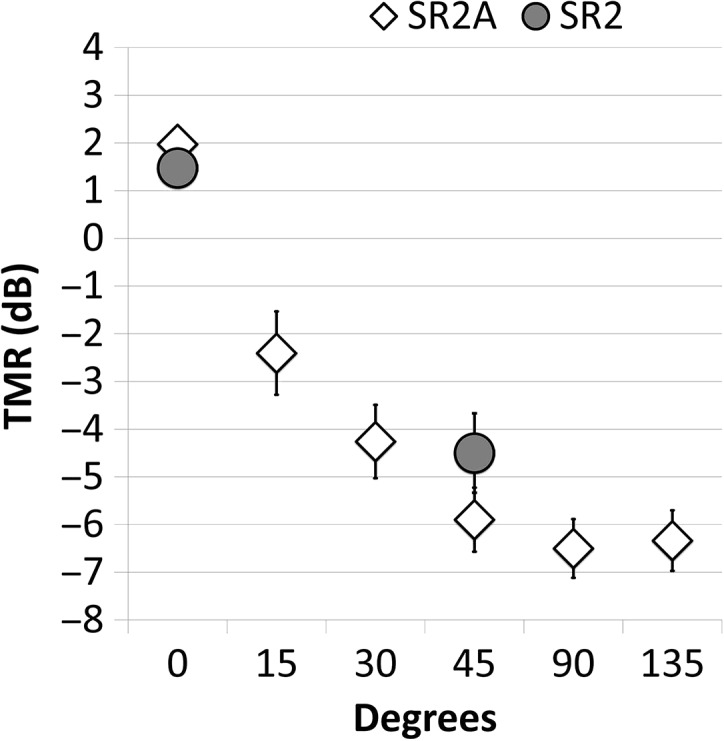
Mean thresholds for the SR2 and SR2A for all spatial separations in Experiment 1. TMR = target-to-masker ratio.

Average performance on the SR2 and the SR2A is shown in Figure 2, and thresholds for all conditions across all repetitions (“runs”) are shown in Table 1. Performance on the SR2A was very similar across all four runs, with colocated thresholds varying between 1.6 and 2.4 dB, and spatially separated thresholds improving with increasing separation, with best performance (an average threshold of −6.5 dB) occurring at separations of 90°. Spatial release also increased steadily with growing spatial separation, with the most release (8.5 dB) being found at 90°. Performance on the SR2 was better for the second repetition in both colocated and separated conditions. For the colocated condition, the two values were similar to those obtained in the SR2A (2.3 and 1.6 dB), but the 45° separation in the SR2A produced better performance (−5.3 to −6.4 dB) than in the SR2 (−3.0 to −5.1 dB).

**Table 1.** Average thresholds and release across conditions for individual runs of both the anechoic chamber (SR2A) and headphone (SR2) versions.

Experiment	Condition	Run	Threshold		Spatial release	
			Mean	Std. deviation	Mean	Std. deviation
SR2A (Anechoic chamber)	0°	1	2.38	1.18		
		2	1.76	1.41		
		3	1.62	1.82		
		4	2.14	1.83		
	15°	1	−2.97	2.60	5.34	2.84
		2	−2.03	3.10	6.90	2.47
		3	−2.69	2.54	8.31	1.91
		4	−1.93	2.84	8.59	3.34
	30°	1	−4.52	2.29	9.03	2.10
		2	−4.76	2.64	3.79	3.20
		3	−3.90	2.35	6.52	3.05
		4	−3.86	3.40	8.17	2.82
	45°	1	−5.93	2.02	8.62	2.40
		2	−6.41	2.21	8.38	2.19
		3	−5.97	2.26	4.31	3.07
		4	−5.28	2.70	5.52	3.02
	90°	1	−6.21	3.53	7.59	2.63
		2	−6.86	1.94	7.90	2.99
		3	−6.28	2.25	7.38	3.61
		4	−6.66	1.91	4.07	3.00
	135°	1	−6.66	1.99	6.00	3.28
		2	−6.62	2.11	7.41	2.96
		3	−5.76	3.03	8.79	2.47
		4	−6.31	1.95	8.45	2.61
SR2 (Headphones)	0°	1	2.34	2.09		
		2	1.59	1.64		
	45°	1	−2.97	3.95	5.31	3.58
		2	−5.10	2.66	6.69	2.42

*Note.* Release is calculated as the difference between the 0° condition and each spatially separated condition for an individual subject on an individual run. *N* = 29.

A repeated-measures analysis of variance (ANOVA, SPSS v. 22) was conducted on the SR2A data, testing the main effects of run and spatial separation. Due to violations of the assumption of sphericity in the data, Greenhouse–Geisser corrections were performed by reducing the degrees of freedom used to calculate the statistical significance. There was a significant effect of separation, *F*(3.36, 107.45) = 273.78, *p* < .001, accounting for 90% of the variance, as estimated by partial eta-squared. There was no significant effect of run or interaction between run and separation (*p* > .05). Pairwise comparisons were used to examine the differences across spatial separations. The colocated 15° and 30° conditions were different from each other and from the larger separations (*p* < .001), whereas the 45°, 90°, and 135° conditions were different from the smaller separations (*p* < .001) but similar to each other (*p* > .05). This pattern can be seen in Figure 2. This same pattern was observed in a repeated-measures ANOVA on SRM, where the effect of separation was significant, *F*(2.7, 75.3) = 85.48, *p* < .001, and accounted for 75% of the variance, but the effect of run was not (*p* > .05), nor was the interaction of run and separation (*p* > .05).

Stepwise multiple linear regression was conducted on the mean data from the SR2A, averaged across all four runs, to examine the effects of age, standard bilateral PTA, and high-frequency PTA on thresholds at each spatial separation (see Table 2 for full statistics). There was no significant modeling result in the colocated condition, but age was the largest potential predictor, accounting for 8% of the variance. Model results showed a significant effect of high-frequency PTA at smaller separations (15° and 30°), accounting for 15% and 36% of the variance. There was also a significant effect of standard bilateral PTA at all larger separations accounting for the following amounts of variance at 45°, 90°, and 135°: 26%, 34%, and 25%, respectively.

**Table 2.** Multiple linear regression statistics for Experiment 1.

Experiment	Spatial separation	Adjusted *R* ^2^	Model statistics	Age		Standard PTA		High-frequency PTA	
				Standard regression coefficient	*p*	Standard regression coefficient	*p*	Standard regression coefficient	*p*
SR2A (Anechoic chamber)	0°	0.082	*F*(1, 28) = 3.510, *p* = .070	0.339	.070	−0.130	.490	0.152	.530
	15°	0.146	*F*(1, 28) = 5.790, *p* = .020	0.080	.730	0.160	.500	**0.420**	**.023**
	30°	0.359	*F*(1, 28) = 16.674, *p* < .001	0.068	.738	0.157	.442	**0.618**	**< .001**
	45°	0.263	*F*(1, 28) = 11.000, *p* = .003	0.199	.237	**0.538**	**.003**	0.183	.401
	90°	0.343	*F*(1, 28) = 15.620, *p* = .001	0.105	.513	**0.605**	**.001**	0.217	.290
	135°	0.245	*F*(1, 28) = 10.070, *p* = .004	0.044	.799	**0.521**	**.004**	0.139	.529
	Release, 15°	0.134	*F*(1, 28) = 5.320, *p* = .029	−0.125	.499	−**0.406**	**.029**	−0.109	.646
	Release, 30°	0.305	*F*(1, 28) = 13.270, *p* = .001	−0.222	.174	−**0.574**	**.001**	−0.309	.174
	Release, 45°	0.298	*F*(1, 28) = 12.871, *p* = .001	−0.028	.865	−**0.568**	**.001**	0.093	.665
	Release, 90°	0.374	*F*(1, 28) = 17.720, *p* < .001	0.081	.604	−**0.629**	**< .001**	0.083	.683
	Release, 135°	0.265	*F*(1, 28) = 9.720, *p* = .004	0.129	.455	−**0.515**	**.004**	0.143	.521
SR2 (Headphones)	0°	0.452	*F*(1, 28) = 24.098, *p* < .001	**0.687**	**< .001**	0.034	.817	−0.014	.940
	45°	0.451	*F*(2, 28) = 12.501, *p* < .001	**0.519**	**.001**	**0.372**	**.015**	−0.162	.529
	Release, 45°	0.232	*F*(1, 28) = 8.145, *p* = .008	−0.187	.288	−**0.481**	**.008**	−0.084	.714

*Note. N* = 29. PTA = pure-tone average.

Stepwise multiple linear regression conducted on the average SRM across all four runs of the SR2A revealed that there was a significant effect of standard bilateral PTA for all spatial conditions tested (see Table 2 for full statistics). At 15°, PTA accounted for 14% of the variance. At 30°, it accounted for 31%. At 45°, the variance accounted for was 30%. At 90° and 135°, the variance accounted for was 37% and 27%, respectively. There were no significant effects of age or high-frequency PTA.

It is worth noting that as spatial separations increased, the amount of variance accounted for by standard bilateral PTA also increased, with the most variance being seen at 90°, perhaps due to the fact that 90° has the largest ITDs and ILDs. At 135°, the variance decreased, perhaps because 135° is similar to a 45° separation.

The differences between the two repetitions of the SR2 were examined using repeated-measures ANOVA. The effect of spatial separation was statistically significant, *F*(1,28) = 189.20, *p* < .001, accounting for 87% of the variance, as estimated by partial eta-squared, as was the effect of run, *F*(1,28) = 16.43, *p* < .001, accounting for 37% of the variance, as estimated by partial eta-squared. The interaction between separation and run was not statistically significant (*p* > .05).

Stepwise multiple linear regression was conducted on TMR for the average of both runs of the SR2 (full statistics shown in Table 2). There was a significant effect of age in the colocated condition, accounting for 45% of the variance. At 45°, there were significant effects both of age and standard bilateral PTA (together accounting for 45% of the variance), which differs from the SR2A results, which found only a significant standard PTA effect at 45° and no age effect. There was no significant effect of high-frequency PTA, which did appear at smaller separations in the SR2A. In regard to SRM, there was a significant effect of standard bilateral PTA, accounting for 23% of the variance, which is similar to the SR2A.

One important question these data can address is the relationships among the conditions tested in the SR2 and the SR2A. Table 3 shows the correlations among age, standard PTA, high-frequency PTA, and the average thresholds in the two conditions of the SR2 and the six conditions of the SR2A for the 29 participants in Experiment 1. Due to the fact that Table 3 contains 39 different correlations, a Bonferroni correction was applied to keep the false-discovery rate at 5%, resulting in a critical *p* value of .05 / 39 = .0013. A number of the relationships survived this correction, including the correlation between the 45° separated conditions in both the SR2 and the SR2A (*r* = .65, *p* < .001), but not the colocated conditions (*r* = .515, *p* = .0043). The 15° and 30° conditions in the SR2A were significantly correlated with both the colocated and separated conditions in the SR2, but the 45° condition in the SR2A was not correlated with the colocated condition in the SR2. In addition, the 90° and 135° conditions in the SR2A were not significantly correlated with either of the SR2 conditions.

**Table 3.** Normalized correlations between headphone (SR2) and anechoic chamber data (SR2A) for Experiment 1.

Test conditions	Standard PTA	High-frequency PTA	Headphones (SR2)		Anechoic chamber (SR2A)					
			0°	45°	0°	15°	30°	45°	90°	135°
Age	0.213	0.642*	0.687*	.598*	0.339	0.317	0.437	0.305	0.230	0.153
Standard PTA		0.649*	0.179	0.483	−0.052	0.366	0.492	0.538	0.605*	0.521
High-frequency PTA			0.433	0.524	0.307	0.420	0.618*	0.455	0.519	0.419
SR2 0°				0.601*	0.515	0.617*	0.662*	0.466	0.340	0.434
SR2 45°					0.355	0.620*	0.644*	0.650*	0.486	0.429

*Note. N* = 29. PTA = pure-tone average.

* *p* < .0013.

Age was correlated with both conditions in the SR2, but none of the conditions in the SR2A. This suggests that there may have been a cognitive aspect to the SR2 that was not present to the same degree in the SR2A. The only significant correlations with PTA were between the standard PTA and the 90° condition of the SR2A and between the high-frequency PTA and the 30° condition of the SR2A. These results were heavily influenced by the conservative significance criterion applied, as the nonsignificant correlations were still fairly large. This suggests that increasing the sample size (or decreasing the number of correlations tested) would have revealed a larger number of significant relationships.

### Discussion

Repetitions of the SR2 and SR2A resulted in mean thresholds that slightly varied across runs, with SR2 thresholds varying slightly more than 2 dB across runs and the SR2A thresholds varying slightly more than 1 dB. Similar threshold values were observed between the SR2 and SR2A in the colocated conditions and between 45° in the SR2 and 30° in the SR2A. This similarity, along with significant correlations between the SR2 at 45° and separations of 15°, 30°, and 45° in the anechoic chamber, supports the use of headphone presentation as a proxy for an anechoic chamber, which is not available to most clinicians.

Stepwise multiple linear regression on the SR2A showed the significance of high-frequency PTA on mid-range separations (15° to 30°) and the significance of standard bilateral PTA on larger separations and SRM. In the SR2, there was a strong age effect in the colocated condition. These results may suggest that at 0°, only the young normal-hearing listeners can achieve lower thresholds, and at small separations (15° to 30°), the ability to use high-frequency information plays a role in improving thresholds. By 45°, older listeners with hearing loss are able to achieve SRM.

One difference between the SR2 and the SR2A was the slightly higher SL used in the SR2 (39.5 vs. 30) and the slightly different SRT estimation techniques. The similarity in results suggests that these differences had little impact on the outcomes. In general, the relationships shown in Table 3 support the findings of the regression analyses and indicate strong relationships between the headphone tests and the anechoic chamber tests, while pointing to some interesting potential directions for future work examining the similarities and differences between real-world cues and similar cues presented in simulated environments.

## Experiment 2

One of the main differences between the SR2 and the SR2A observed in Experiment 1 was the 2-dB difference in separated thresholds observed in the SR2 between runs 1 and 2. One possibility is that it reflects a genuine difference in repeatability between the two measures. Another possibility is that 27 of the 29 participants had already run the SR2 before being tested on the SR2A. To try to distinguish between these possibilities, in the second experiment, data from eight runs of the SR2 were examined for 40 participants, 16 of whom were also participants in Experiment 1.

### Subjects

**Figure 3.**
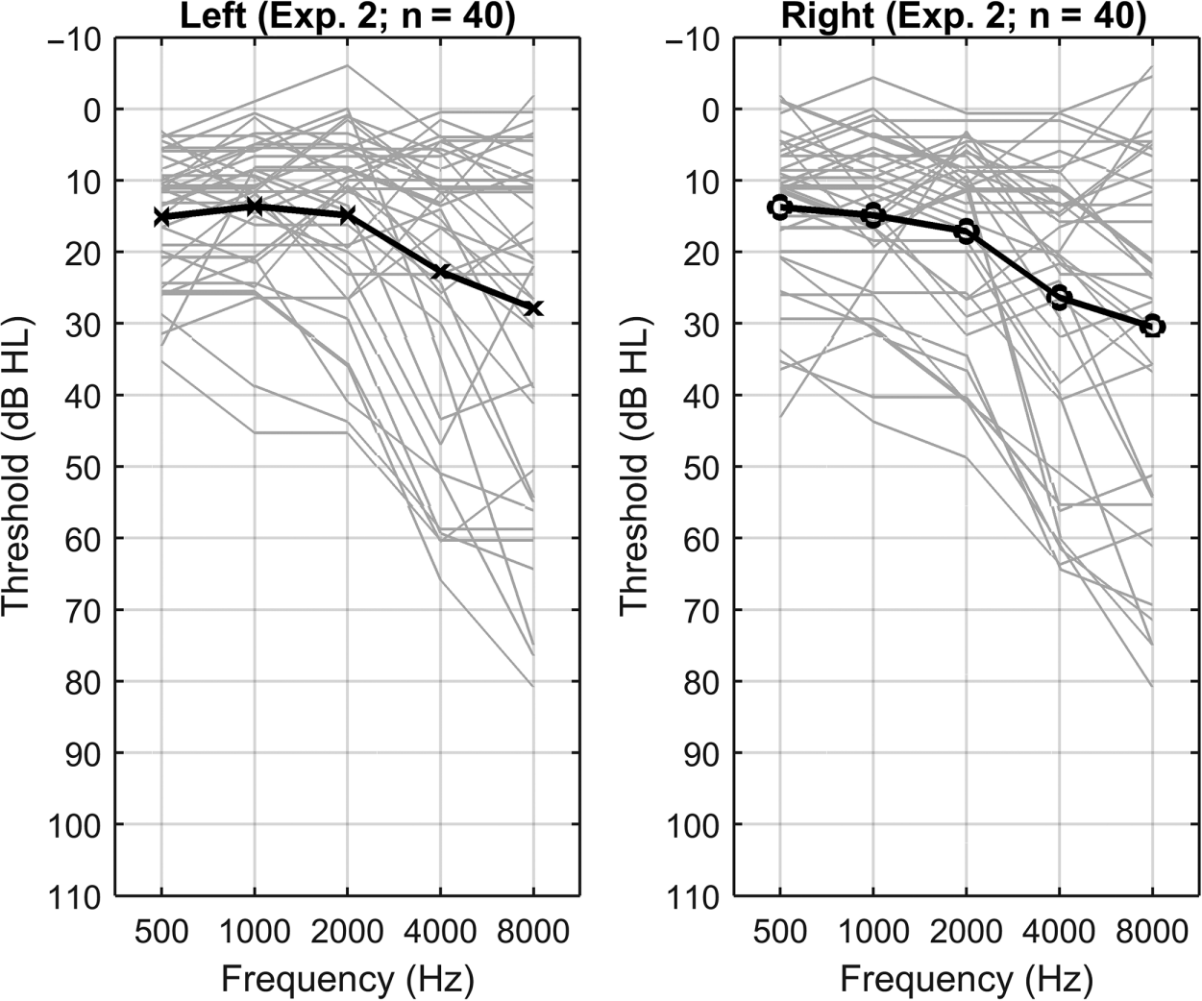
Mean (black lines) and individual (gray lines) audiometric thresholds for the 40 individuals from Experiment 2.

All participants within the age range of 22–79 years (mean 55.35 years) had bilateral PTAs (0.5, 1, 2, and 4 kHz) below 41 dB HL (mean 14.90 dB ± 9.93 dB HL) and high-frequency PTAs (2, 4, and 8 kHz) below 60 dB HL (mean 23.25 dB ± 17.26 dB HL). All had fairly symmetrical hearing at 2 kHz and below (most had differences between the ears of less than 10 dB at all frequencies, and no listeners had differences exceeding 20 dB). SRT values ranged from 3 to 43 dB HL. Average audiograms are shown in Figure 3. All listeners were in good health with no history of otological disorders and had scores of 24 or higher on the Mini-Mental State Examination (Folstein et al., 1975), and none of the individuals used hearing aids.

### Results

**Figure 4.**
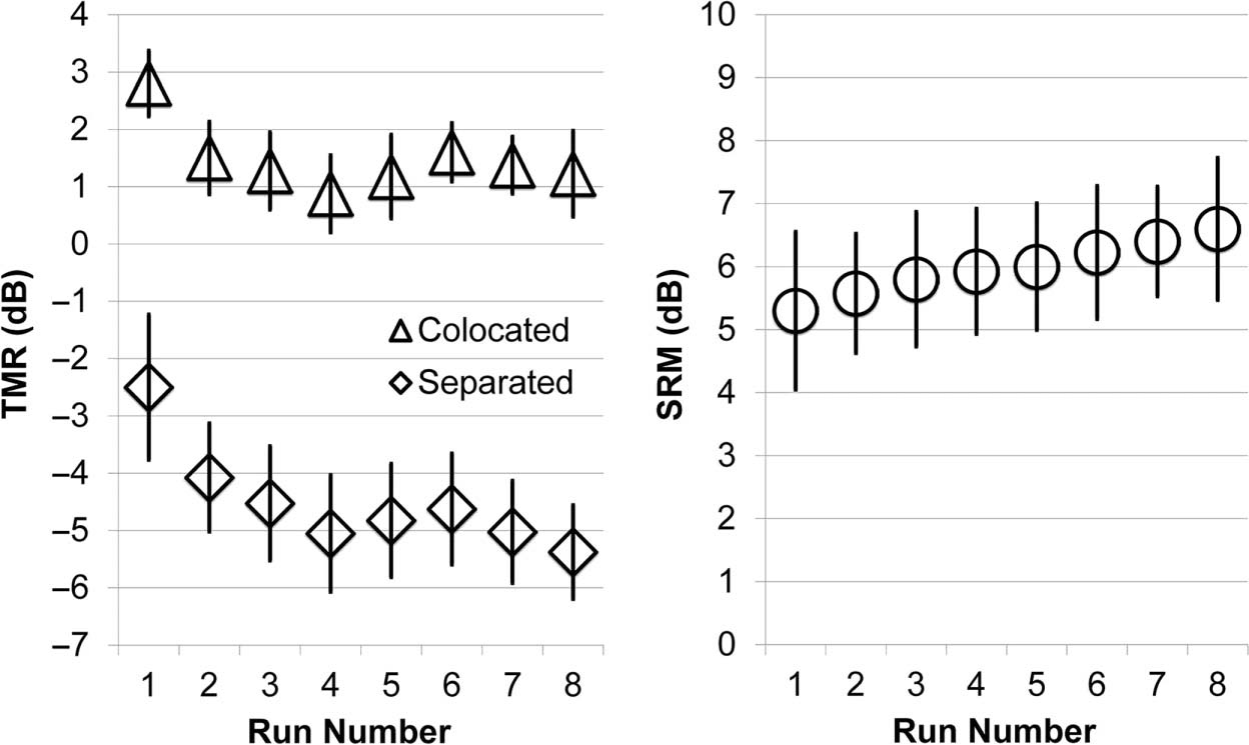
Mean thresholds and spatial release from masking (SRM) across the eight runs of the SR2 in Experiment 2. TMR = target-to-masker ratio.

Figure 4 and Table 4 show the thresholds for all participants across eight runs. The mean threshold for the colocated condition was 1.48 dB, which is similar to, but slightly below, the best performance seen in the colocated condition in the SR2A. The mean threshold for the spatially separated condition was −4.5 dB, which is worse than SR2A thresholds at 45° but similar to the SR2A thresholds at 30° and to the thresholds for the SR2 observed in Experiment 1. The mean amount of SRM was 5.98 dB, which is also similar to what was seen in Experiment 1 for the SR2 and for the SR2A at 30°. As can be seen in Figure 4, the highest values always occurred at run 1, after which the values were within 1.5 dB of each other.

**Table 4.** Average thresholds and release across conditions for eight runs of the headphone (SR2) test for Experiment 2.

		Threshold		Spatial release	
Condition	Run	Mean	Std. deviation	Mean	Std. deviation
0°	1	2.80	1.81		
	2	1.50	2.00		
	3	1.28	2.14		
	4	0.88	2.13		
	5	1.18	2.31		
	6	1.60	1.61		
	7	1.38	1.58		
	8	1.23	2.37		
45°	1	−2.50	4.01	5.30	3.96
	2	−4.08	2.98	5.58	2.99
	3	−4.53	3.15	5.80	3.38
	4	−5.05	3.22	5.93	3.14
	5	−4.83	3.13	6.00	3.18
	6	−4.63	3.06	6.23	3.34
	7	−5.03	2.82	6.40	2.75
	8	−5.38	2.59	6.60	3.56

*Note.* Release is calculated as the difference between the 0° condition and spatially separated conditions for an individual subject on an individual run. *N* = 40.

To analyze the sources of variability in thresholds for the SR2, stepwise multiple linear regression was conducted to examine the effects of age, PTA, and high-frequency PTA on TMR. There was a significant effect of age in the colocated condition, accounting for 27% of the variance, but no effect of standard PTA or high-frequency PTA (see Table 5 for full statistics). For the 45° condition, there was a significant effect of standard bilateral PTA, accounting for 47% of the variance, but no significant effects of high-frequency PTA or age. Table 5 also shows the results of stepwise multiple linear regression relating the same factors to SRM in the SR2. There was a significant effect of standard PTA, accounting for 45% of the variance, but there was no significant effect of high-frequency PTA (*p* = .42) or age (*p* = .97).

**Table 5.** Multiple linear regression statistics for Experiment 2.

Spatial separation	Adjusted *R* ^2^	Model statistics	Age		Standard PTA		High-frequency PTA	
			Standard regression coefficient	*p* value	Standard regression coefficient	*p* value	Standard regression coefficient	*p* value
0°	0.271	*F*(1, 39) = 15.464, *p* < .001	**0.538**	**< .001**	−0.107	.506	−0.076	.676
45°	0.467	*F*(1, 39) = 35.191, *p* < .001	0.247	.068	**0.693**	**< .001**	0.356	.099
Release, 45°	0.445	*F*(1, 39) = 32.255, *p* < .001	−0.005	.973	−**0.678**	**< .001**	−0.180	.421

*Note. N* = 40. PTA = pure-tone average.

With the purpose of examining all eight runs, repeated-measures ANOVA was conducted on TMR in which the factors of run and spatial separation were tested. The main effect of separation was statistically significant, *F*(1, 39) = 250.61, *p* < .001, and accounted for 87% of the variance in TMR. The main effect of run was also significant, *F*(7, 273) = 11.35, *p* < .001, and accounted for 23% of the variance. The interaction between run and separation was not significant (*p* > .05). Pairwise comparisons between runs were conducted with a Bonferroni correction for multiple comparisons to further examine the main effect of run. Differences across runs were found to be greatest for run 1 (mean of 1.3-dB difference for colocated and 2.0-dB difference for spatially separated [*p* < .01]), and mean differences for the other runs were no greater than 1 dB and were not significantly different from each other (*p* > .05). The effect of run on SRM is also illustrated in Figure 4 and was examined with repeated-measures ANOVA, revealing no significant differences among runs (*p* > .05). However, the visible upward trend in the release of 0.1 to 0.2 dB per run (1.5-dB increase from run 1 to run 8) is supported by the finding of a significant linear relationship (*p* < .01) with increasing run number, accounting for 16% of the variance in SRM.

**Figure 5.**
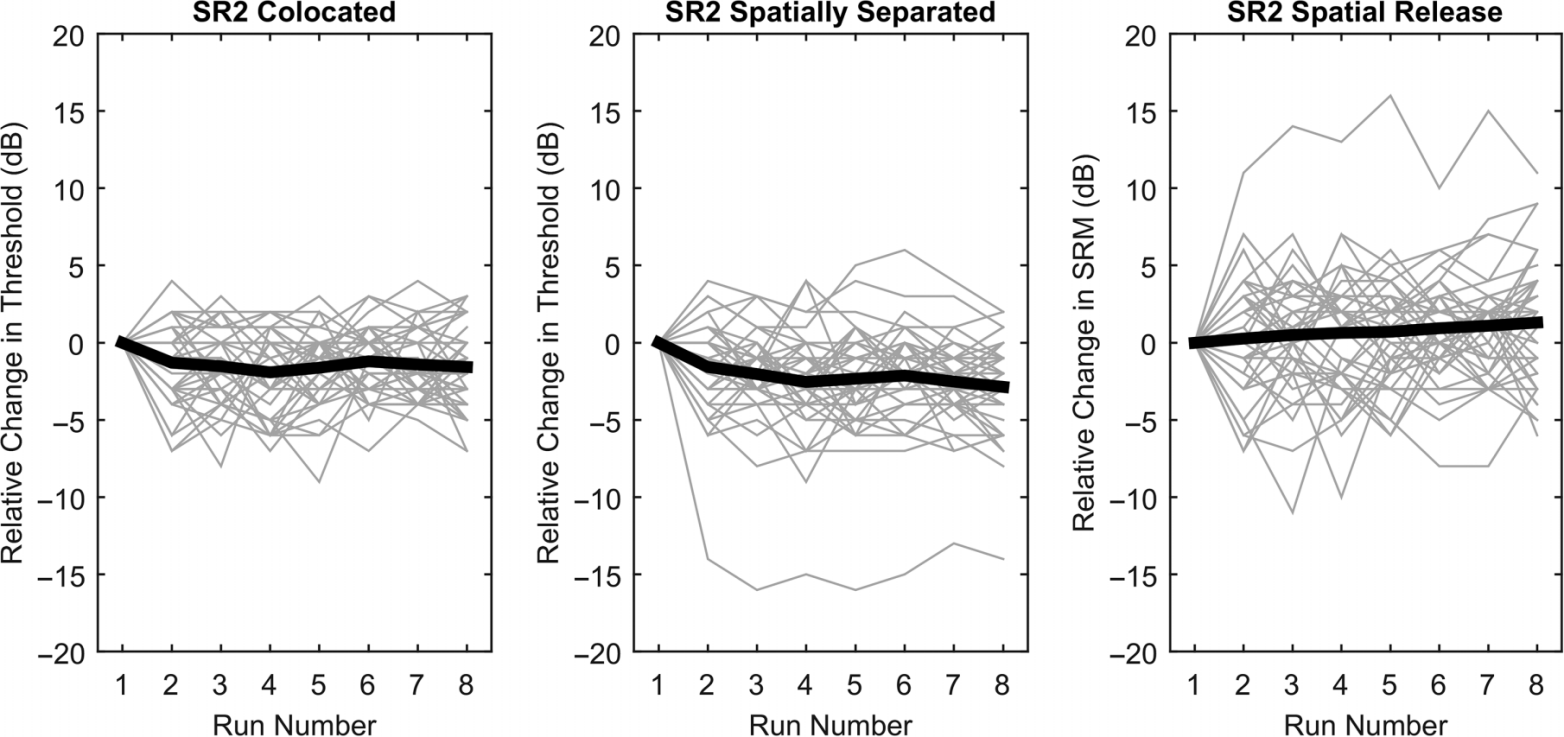
Mean (black lines) and individual (gray lines) differences between the first run and the subsequent seven runs in Experiment 2, plotted for the colocated and separated thresholds and for spatial release from masking.

The relative changes in threshold across runs for each of the 40 participants are illustrated in Figure 5, where the left and middle panels show the difference in threshold between run 1 and the subsequent runs for the colocated and separated conditions, and the right panel shows the differences for SRM. The individual listeners are each represented by gray lines, and the mean differences are plotted as black lines. In the colocated condition, the mean absolute value of the difference between the first run and the subsequent runs (the “relative change”), averaged across listeners, was 2.00 dB (*SD* = .98 dB). For the separated condition, the average relative change was 2.55 dB (*SD* = 2.06 dB). For SRM, the average relative change was 2.70 dB (*SD* = 1.65 dB). It can be seen that there was only a single listener with a very large relative change, indicated by the negative values below −10 in the middle panel and the values greater than 10 in the right panel. Even this listener did not show systematic changes for runs 2 to 8, however.

## General Discussion

The two experiments described above demonstrate that the SR2 is a reliable method of evaluating speech-on-speech masking and SRM. Experiment 1 revealed strong relationships between headphone and anechoic chamber testing. Experiment 2 revealed small differences across repeated testing for the same 40 participants.

Age effects were found in the colocated conditions of the SR2 but not the spatially separated conditions, which is consistent with Srinivasan et al. (2016) who found that with little or no separation, younger listeners may achieve better thresholds and SRM compared to older listeners with similar hearing thresholds. In Jakien et al. (2017), there were no significant effects of age on thresholds or SRM examined at 45°. However, the large sample included many with moderate hearing loss, and the general trend in that study was that thresholds worsened as age increased.

For 15° and 30° in Experiment 1, the current study found an effect of high-frequency PTA, which agrees with the work of Marrone et al. (2008) and supports the work of Jakien et al. (2017), Besser et al. (2015), and Strelcyk and Dau (2009), suggesting that NH listeners can use information in the high-frequency region of the speech stimuli to improve performance in the presence of speech maskers, perhaps by “glimpsing” the target (Ahlstrom et al., 2014; Brungart & Iyer, 2012; Glyde, Buccholz, Dillon, Best, et al., 2013). These results are also consistent with the findings of Glyde, Buchholz, Dillon, Cameron, and Hickson (2013), supporting the importance of high-frequency ILDs for SRM. It is also possible that a combination of glimpsing and high-frequency ILDs provides access to spatial cues that are more easily detected than at lower frequencies where there is greater overlap of target and masker spectra. Future work should address the specific possibility of binaural glimpsing in high-frequency regions, as well as the possibility that hearing impairment reduces the ability to benefit from glimpses (Best et al., 2016, 2017).

Experiment 1 showed that the 45° thresholds of the SR2 were best correlated to 45° thresholds for the SR2A, although performance at 45° over headphones was most similar to that obtained in the anechoic chamber at 30°. Perhaps the better performance obtained at 45° in the anechoic chamber was due to the ability of the participants to make small head movements, which improved the perceived separation of the talkers. Another possibility is that the nonindividualized head-related transfer functions in the SR2 did not provide as compelling spatial percepts as were obtained in an actual spatial environment. Regardless of these small differences in absolute performance, the strong correlations between the SR2 and SR2A and the similarity of performance show that a virtual array can accurately predict thresholds produced in an anechoic chamber.

SR2 thresholds in Experiment 2 varied by 2–3 dB across eight runs of the test, with the only systematic change being a slight deviation in thresholds occurring from the first to the second run, which may have been due to a learning effect. This information helps determine how a clinical version of the test could be implemented. If a patient's thresholds for one run were within a normal range, no other runs of the test would be necessary. However, if the patient's thresholds were close to or within the abnormal range, it would be prudent to run the test two or more times and average the results. Clinically, the SR2 provides an accurate depiction of who may struggle with speech in noise and who might, with additional testing, be revealed to have a central auditory processing deficit. The automated nature of the SR2 allows it to function as a rapid screening test for speech-on-speech masking ability that could be used on large numbers of patients with little impact on the time available in a busy clinical environment. Work is ongoing to determine normative data for SR2 and to make it available as a freely available application that could be run using a tablet or a phone. One advantage of having a fast, reliable, automated measure of SRM is the ability to gather larger data sets with greater distributions of age and hearing in an efficient and accurate way. It would be useful to extend these findings to other research projects, such as examining age and hearing loss at small separations.

In summary, we have shown that the SR2 is a reliable and useful test of SRM. We have demonstrated the consistency of the test across several runs and have shown how accurately thresholds over headphones represented thresholds in an anechoic chamber. We have examined how hearing loss and age impact release from masking and are currently working on the free application version of the SR2 to be used in the laboratory and clinic.
